# Biocrust morphogroups provide an effective and rapid assessment tool for drylands

**DOI:** 10.1111/1365-2664.12336

**Published:** 2014-10-01

**Authors:** Cassia F. Read, David H. Duncan, Peter A. Vesk, Jane Elith

**Affiliations:** ^1^ School of Botany University of Melbourne Parkville Vic. 3010 Australia; ^2^ Department of Environment and Primary Industries Arthur Rylah Institute for Environmental Research 123 Brown St Heidelberg Vic. 3084 Australia

**Keywords:** biological soil crust, bryophyte, cyanobacteria, ecological integrity, functional group, lichen, morphological group, multivariate regression trees, rapid survey, semi‐arid

## Abstract

1. Biological soil crusts (biocrusts) occur across most of the world's drylands and are sensitive indicators of dryland degradation. Accounting for shifts in biocrust composition is important for quantifying integrity of arid and semi‐arid ecosystems, but the best methods for assessing biocrusts are uncertain. We investigate the utility of surveying biocrust morphogroups, a reduced set of biotic classes, compared to species data, for detecting shifts in biocrust composition and making inference about dryland degradation.
2. We used multivariate regression tree (MRT) analyses to model morphogroup abundance, species abundance and species occurrence data from two independent studies in semi‐arid open woodlands of south‐eastern Australia. We advanced the MRT method with a ‘best subsets’ model selection procedure, which improved model stability and prediction.
3. Biocrust morphogroup composition responded strongly to surrogate variables of ecological degradation. Further, MRT models of morphogroup data had stronger explanatory power and predictive power than MRT models of species abundance or occurrence data. We also identified morphogroup indicators of degraded and less degraded sites in our study region.
4. *Synthesis and applications*. Sustainable management of drylands requires methods to assess shifts in ecological integrity. We suggest that biocrust morphogroups are highly suitable for assessment of dryland integrity because they allow for non‐expert, rapid survey and are informative about ecological function. Furthermore, morphogroups were more robust than biocrust species data, showed a strong response to ecological degradation and were less influenced by environmental variation, and models of morphogroup abundance were more predictive.

## Abstract 2

Sustainable management of drylands requires methods to assess shifts in ecological integrity. We suggest that biocrust morphogroups are highly suitable for assessment of dryland integrity because they allow for non‐expert, rapid survey and are informative about ecological function. Furthermore, morphogroups were more robust than biocrust species data, showed a strong response to ecological degradation and were less influenced by environmental variation, and models of morphogroup abundance were more predictive.

## Introduction

Dryland ecosystems encompass *c*. 40% of the earth's terrestrial surface, house nearly one‐third of the human population and are under intense pressure from changing land‐use practices and climate (Mortimore 2009). For decades, ecologists have highlighted the importance of biological soil crusts (biocrusts) in dryland management (Brotherson, Rushforth & Johansen 1983; Bowker 2007). Biocrusts are complex communities of soil biota, typically dominated by bryophytes, lichens, cyanobacteria and algae. They are found in nearly all dryland biomes of the world where aridity decreases competition from vascular vegetation and soils are sufficiently stable (Büdel 2003). Biocrusts are indicators of highly functional ecosystems (Maestre *et al*. 2012). While their value as ecological indicators is recognized in several standard rangeland survey techniques that record total biocrust cover (Pellant *et al*. 2000; Tongway & Hindley 2004), the best methods to assess biocrusts are uncertain.

Consideration of biocrusts in dryland management is motivated and justified by the relationship between the biocrust layer and the ecological integrity of sites where they naturally occur. In this context, integrity refers to structure, function and composition compared to a natural or historical range of variation (*sensu* Tierney *et al*. 2009). Biocrusts directly affect three components of integrity: biocrusts of different morphologies can dominate ground cover and strongly influence structure of arid and semi‐arid ecosystems where vascular plant cover is low (Belnap & Lange 2003); biocrusts are functionally important for soil stability (Chaudhary *et al*. 2009), soil hydrology (Chamizo *et al*. 2011), nutrient cycling (Zhao, Xu & Belnap 2010) and vegetation recruitment (Su *et al*. 2009); and finally, the species composition of the biocrust influences its function (Bowker *et al*. 2011). Further, biocrusts are highly sensitive to physical disturbances such as livestock trampling, and early signals of ecosystem degradation include loss of biocrust cover and simplification (Bowker 2007), while increases in cover indicate ecosystem recovery (Read *et al*. 2011).

Assessment of biocrusts is neither easy nor cheap, so efficiency is important. Rapid‐survey methods that capture shifts in biocrust composition in response to degradation would be valuable for managers wanting to assess the ecological integrity of dryland sites in relation to reference conditions (i.e. minimally disturbed reference sites), monitor changes in site integrity over time or determine ecosystem states for state‐and‐transition models. Surveying biocrusts is challenging: biocrust communities are often diverse, and component species are small and difficult to identify, with much of their taxonomy still unresolved. This leads to uncertainty about which attributes of biocrusts to measure in order to detect change. A particular difficulty in choosing a survey method is the limited understanding of trade‐offs between detailed‐and‐slow and coarse‐and‐rapid surveys. Total biocrust cover is relatively quick and simple to assess and is informative about soil erodibility (Belnap & Gillette 1997), but overlooks other important attributes of ecological integrity. For instance, biocrust composition and function can vary widely with successional age or land use, without a corresponding change in total cover (Chamizo *et al*. 2011). While species assessments have revealed changes in biocrust composition in response to degradation (Muscha & Hild 2006; Lalley & Viles 2007), these assessments can be painstakingly slow, require highly trained expertise and are therefore impractical for rapid surveys. Further, it can be difficult to generalize about shifts in species composition.

The morphological group (morphogroup) classification of Eldridge & Rosentreter (1999) represents the best rapid‐survey method available for both recording biocrust composition and making inference about biocrust structure and function. Other rapid methods classify biocrusts by successional stage (Dougill & Thomas 2004; Belnap *et al*. 2008), but these coarse levels are probably invariant across ecological and disturbance gradients, except between environmental extremes. Ideally, rapid surveys of biocrusts would measure the community composition of functional traits (*sensu* Violle *et al*. 2007), enabling generalization of species’ response to degradation and providing insight into the effect of these changes on ecosystem processes. Unlike the field of seed plant ecology where these concepts are well developed (Díaz *et al*. 2004), functional traits have been largely overlooked for organisms comprising the biocrust, except for difficult‐to‐measure effect traits (Cornelissen *et al*. 2007). In the absence of coherent knowledge about biocrust species traits, Eldridge & Rosentreter (1999) argue that biocrust species’ morphology largely determines their function; hence, they classify species into simple morphological classes (e.g. short or tall moss, crust‐like or leafy lichen). In this sense, morphogroups are similar to categorical functional effect traits (*sensu* Violle *et al*. 2007). Several studies support this view, with evidence for functional differences between morphogroup effects on vegetation recruitment (Su *et al*. 2009), water infiltration (Maestre *et al*. 2002) and soil stability (Jimenez Aguilar *et al*. 2009 *)*. Differences between the functional responses of morphogroups to degradation have also been shown (Eldridge & Koen 1998; Muscha & Hild 2006). However, the utility of morphogroups for rapid ecological surveys has not been directly evaluated, and the degree to which morphogroups summarize and generalize biocrust species responses to degradation is not yet demonstrated.

If morphogroups do capture species responses, they represent a valuable rapid assessment tool for measuring and generalizing biocrust response to degradation and for informing land managers about changes in ecosystem integrity. But how do we establish confidence in a reduced set of biotic classes such as morphogroups? This is a long‐standing question in applied ecology when choosing a rapid‐survey method that uses a higher level of biotic classification (Osborne, Davies & Linton 1980; McIntyre, Lavorel & Tremont 1995; Bunce *et al*. 2008). For example, River Invertebrate Prediction and Classification System (RIVPACS) uses macroinvertebrate composition as an indicator of the biotic quality of freshwaters. The appropriate level of taxonomic resolution has been an ongoing concern for developers of the method (Wright, Sutcliffe & Furse 2000). Simplification of species data into a reduced set of classes undoubtedly increases the ease and speed of biotic surveys, but it is critical to know the information cost of simplification, before choosing an appropriate survey resolution.

The purpose of this study was to investigate the utility of different survey resolutions for assessing shifts in biocrust composition, and in particular the utility of biocrust morphogroups (Eldridge & Rosentreter 1999). We focus on the response of biocrust composition to ecological degradation (including livestock trampling and landscape fragmentation) because biocrust composition influences its structure and function and therefore encompasses the three components of ecological integrity. We used data from two previous studies of a semi‐arid, dryland agricultural landscape in south‐eastern Australia (Read *et al*. 2008, 2011). We analysed response of biocrust communities as measured at three resolutions – biocrust morphogroup abundance, species abundance and species occurrence – to ecological variables and evaluate their utility in terms of ease of data collection and information gained. Given that morphogroups allow for non‐expert, rapid survey and they are informative about ecological function, we judged them to be more useful than species data if they showed a strong response to measured variables and if they accurately captured component species’ response to ecological degradation. We used multivariate modelling methods to investigate biocrust community response at each level of resolution and to identify variables explaining community composition from a large selection of candidate variables. Specifically, we asked: (1) Which variables explain biocrust composition, measured at the species (occurrence and abundance) and morphogroup level? (2) Do morphogroups show as strong a response to degradation variables as species, such that morphogroups are useful for summarizing and generalizing species’ responses? (3) Can we identify biocrust morphogroup indicators of degraded ecosystems? Our results will be useful for land managers who make decisions aimed at halting or reversing the loss of ecological integrity in dryland landscapes.

## Materials and methods

The two data sets analysed in this paper have different sampling methodologies and arose from two separate, published studies on biocrusts in the dryland agricultural zone of north‐west Victoria, Australia. The first data set (hereafter ‘fragmentation study’) was collected in 2005 to investigate variables explaining biocrust abundance in a fragmented landscape (Read *et al*. 2008). The second data set (hereafter ‘fencing study’) was collected in 2006 to investigate recovery of biocrusts following livestock exclusion (Read *et al*. 2011). The fencing study covered a restricted rainfall gradient (370–410 mm mean annual rainfall) within the fragmentation study region (330–440 mm mean annual rainfall). For each study, we measured potential explanatory variables of biocrust abundance in the field, in the laboratory and from geographic information system (GIS) data (Table 1) to understand variation in biocrust composition. A brief outline of methodology for each study is provided below.

**Table 1:** Candidate variables used in multivariate regression tree analyses, with scale of measurement (remnant patch, 20‐m^2^ quadrat or 0·5‐m^2^ quadrat) and summary statistics (mean and min/max in parentheses) for each measured variable

Variable	Scale	Mean and range	
		Fragmentation study	Fencing study
Bioregion	Patch	Calcareous dunes and alluvial plains	Alluvial plains
Remnant patch size	Patch	Small (0·5–5 ha), medium (5–10 ha), large (>20 ha)	–
Time since fencing (years)	Patch	–	15·6 (1–50)
Vegetation community	Patch	–	Blackbox, buloke and mallee
Thorium and potassium (Th/K) ratio^a^	Patch	4·3 (3·6–5·4)	4·4 (3·8–5·0)
Available P (mg kg^−1^)	20 m^2^	19·1 (4–98)	16·2 (5–43)
Grazing intensity^b^	20 m^2^	(Low, medium, high)	(low, medium, high)
Location in remnant	20 m^2^	Windward (west) edge, centre, leeward edge	–
Organic soil C (%)	20 m^2^	1·8 (0·48–5·0)	2·4 (1·3–3·4)
pH (H_2_0)	20 m^2^	7·7 (6·2–8·9)	7·0 (6·1–8·5)
Total soil N (%)	20 m^2^	0·11 (0·02–0·30)	0·19 (0·11–0·30)
Tree (proportion)	20 m^2^	0·36 (0–0·85)	–
Exotic annual (proportion)	0·5 m^2^	0·23 (0–0·87)	0·18 (0–1·0)
Native perennial grass and shrub (proportion)	0·5 m^2^	0·10 (0–0·39)	–
Native perennial grass (proportion)	0·5 m^2^	–	0·13 (0·00–0·35)

a Remotely sensed radiometric signal (minimum values associated with heavy clay soil, maximum values associated with sandy loam); calculated as (Thmax−Th)/K.

b Grazing intensity scored as: low – no or little evidence of biomass removal, herbivore dung or disturbance by hooves; medium – localized signs of grazing, some dung and soil disturbance, tussock structure and understorey biomass moderate; and high – extensive, homogenous biomass removal, considerable dung and soil disturbance.

### Field Methods

#### Fragmentation study field methods

We sampled 25 remnant patches of vegetation (patches) across the study region in a stratified random design. Patches were stratified across three size classes and two dominant soil–vegetation associations: alluvial plains with grassy woodlands dominated by either *Eucalyptus largiflorens* or *Allocasuarina luehmannii* and calcareous dunes with woodlands dominated by multistemmed ‘mallee’ *Eucalyptus* spp. We sampled each patch at three locations with 20 × 20 m quadrats; locations were in the remnant interior (centre), the windward (west) edge and the leeward (east) edge. Three patches were so small that only one or two sampling locations could be assessed. Within each quadrat, we recorded the projected cover of trees, collected soil cores for soil analysis and subsampled total biocrust, biocrust morphogroup, shrub, perennial grass, litter and exotic annual cover in ten 0·5 × 0·5 m quadrats. Biocrust species were collected from each small quadrat for later identification. Mean cover of each life‐form was calculated for each larger quadrat at each patch, so the 710 small quadrats were pooled to 71 quadrats.

#### Fencing study field methods

We sampled 21 remnant patches (<30 ha in size) where livestock had been excluded by fencing for a known period of time (1 to *c*. 50 years). Sampling focused on the alluvial plains (see above); however, some ‘mallee’ vegetation, characteristic of the calcareous dunes, was included. We sampled each patch at two haphazardly selected locations >150 m from an edge. At each location, we collected and bulked five soil cores for analysis and recorded shrub and grass cover within three 0·5 × 0·5 m quadrats that were stratified to capture three microenvironments representing variation in sun exposure due to shading from canopy trees. We recorded the cover of each biocrust species in a 0·25 × 0·25 m subquadrat within each small quadrat. Where species cover was difficult to assess due to species intermixing, we subsampled again using three 9‐cm‐diameter cores and estimated mean cover of each species under the microscope. Samples of each biocrust species from each subquadrat were collected for later identification/confirmation. Mean cover (%) of each life‐form was calculated for each microenvironment in each patch (i.e. across the two locations), so the 126 small quadrats sampled were pooled to 63 quadrats.

### Species and Morphogroup Data

Species were identified to the lowest taxonomic level possible for both studies. Where species could not be confidently identified, they were assigned to operational taxonomic units, and where genera could not be split confidently to the species level, they were grouped into representative species groups. For simplicity, taxonomic units will be referred to as species. We estimated morphogroup cover in the field for the fragmentation study and in the laboratory for the fencing study by assigning each species to a morphogroup and summing cover of all species within each morphogroup. Species frequencies and their assignment to morphogroups are provided (See Appendix S1 in Supporting information). We estimated from our field and laboratory experience that surveying biocrust composition and abundance in an average (0·5 × 0·5 m) quadrat takes 10 min for biocrust morphogroups and 90 min for species in our study region (C. Read, pers. obs). Species surveys were time‐consuming because of high moss diversity and intermixing of visually similar species (requiring subsampling and cover estimation with a binocular microscope) and because mosses usually lacked fertile parts, requiring observation of cell structure with a slide microscope to confirm species identity.

### Data Analysis

#### Data preparation

Prior to analysis, we removed quadrats with no biocrust recorded, leaving 61 and 52 quadrats from the fencing and fragmentation studies, respectively. We also removed rare species (occurring in <5% of sites) from the data set as rare species often occur haphazardly and can increase noise (McCune & Grace 2002). We then log‐transformed the cover data (*f*(*x*) = log(*x* + 1)) to compress high values and spread low values, an appropriate transformation for our data which had a high degree of variation (variance > mean), many zeros and a few very abundant species (Clarke & Green 1988). Finally, we standardized data by quadrat total cover to equalize the influence of quadrats with high and low total biocrust cover and to improve detection of changes in species and morphogroup dominance.

Relevant habitat and environmental variables were identified in previous studies (Read *et al*. 2008, 2011; summarized in Table 1). While nitrate was of interest to our study, we excluded nitrate data from analyses because our field sampling regime and length of field trips meant that we could not preserve samples adequately for reliable estimation of nitrate concentrations.

To aid interpretation of results and discussion, we identified variables most plausibly linked to ecological degradation, based on a related study from the region (Duncan *et al*. 2007). *Degradation variables* were as follows: remnant patch size (remnant size is negatively correlated with exposure to livestock trampling); location in remnant (proximity to the windward/western edge is associated with exposure to sediment dumping); time‐since‐livestock exclusion; grazing intensity; and exotic annual cover.

#### Multivariate regression trees (MRTs)

The aim of our analyses was to investigate explanatory variables of biocrust composition measured at the fine‐scale species level and the coarse‐scale morphogroup level and to explore the utility of morphogroups for assessing ecological integrity. Our response variables were species occurrence (presence/absence) and morphogroup abundance (proportion cover) for both the fencing and the fragmentation studies and additionally species abundance (proportion cover) for the fencing study. We analysed each unique response/survey data set individually. The same candidate predictor set comprising habitat and degradation variables was used across models (Table 1), allowing us to investigate biocrust response to degradation in the context of important environmental gradients and different habitat conditions.

We used MRTs to group quadrats with similar community composition and to define the ecological characteristics of each group (De'ath 2002). MRTs are a constrained analysis that repeatedly splits the assembled set of quadrat data into two groups that represent a distinct community composition, with each group defined by associated environmental values. MRTs are useful because they allow development of predictive models and analysis of complex ecological data, including missing values, nonlinear relationships between variables and co‐linearity between explanatory variables. Specifically, we used the sum‐of‐squares MRT described by De'ath (2002) and available in r (version 3.0.0; package ‘mvpart’ version 1.6‐0). Hereafter, all description refers to that specific type of MRT. For a more detailed account of the MRT method, see Appendix S2 (Supporting information).

With MRTs, we need methods for model selection. The relative error (RE) of an MRT is the final error relative to the error of the initial, unsplit tree and is the inverse of its explanatory power or *R* ^2^. The cross‐validated relative error (CVRE) was used to select the best MRT, representing the capacity of the tree to predict community composition for new quadrats. CVRE = 0 indicates perfect prediction, and CVRE ≥1 indicates no predictive power. For this study, we describe a CVRE <0·60 as strong predictive power.

Modelled observations of species abundance and morphogroup abundance were not independent, but hierarchically structured, with pooled, replicate‐quadrat estimates nested within sites for both studies. We dealt with this structure by appropriately organizing our cross‐validation procedure (Fabricius & De'ath 2008; details in Appendix S3, Supporting information).

During model development, we discovered that – depending on the candidate set of predictors – model fitting could be ‘locked’ into building a tree with less than optimal predictive performance for the data set. We addressed this issue and developed code to fit many models on subsets of predictors, to identify the most predictive (best) model. We call this ‘best subsets model selection’. Further details of this issue and model code for our procedure are provided in Appendix S4 (Supporting information).

#### Null models

We constructed null models to investigate whether results of morphogroup analyses were somehow associated with modelling a reduced number of entities (morphogroups compared with species). The results (Appendix S5, Supporting information) satisfied us that the estimated models were behaving sensibly.

#### Indicator morphogroup and species analysis

To identify morphogroup indicators of site degradation, we used the [‘mvpartwrap’ (Marie‐Helene Ouellette with Contributions from Pierre Legendre 2012), R package (version 0.1‐9) (R Core Team 2013)] where indicators are based on morphogroup fidelity to and abundance within leaves of the MRT.

## Results

Sixty species were recorded in the fragmentation study, including 27 mosses, 28 lichens and 5 liverworts; these were grouped into six morphogroups. Thirty‐nine of these species were relatively common (>5% of the 52 quadrats containing biocrusts) and used in the multivariate analyses of species data. Fifty‐four ‘species’ were recorded in the fencing study, including 27 moss, 21 lichen, 5 liverwort species and black crust (cyanobacterial crust), and these were grouped into ten morphogroups. Of these, 24 were relatively common (>5% of the 61 quadrats containing biocrust) and used in multivariate analyses of species data. The *Gemmabryum pachytheca* group was the most common ‘species’ in the fragmentation study (frequency = 62·3%). *Triquetrella papillata* was the most common species (frequency = 69·8%) in the fencing study and occurred at the highest abundance across sites (mean abundance = 20·9%). Detailed species information (including assignment to morphogroups) is provided in Appendix S1 (Supporting information). To our knowledge, this is the first time that such information has been recorded for this region.

### Biocrust Community Composition

**Figure 1:**
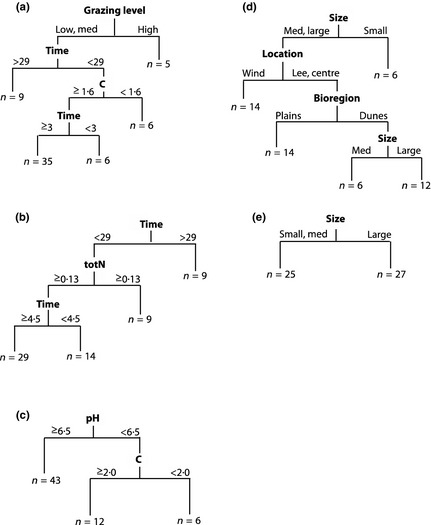
Multivariate regression trees (MRTs) for two studies, comparing two to three levels of data resolution. Trees shown are those with the lowest cross‐validated relative error (CVRE) of all possible trees compared through a *best subsets* procedure. MRTs are as follows: mean morphogroup cover (a, relative error (RE) = 0·48, CVRE = 0·67), species occurrence (b, RE = 0·81, CVRE = 0·96) and mean species cover (c, RE = 0·71, CVRE = 0·80) per site for the fencing study (*n* = 61); and mean morphogroup cover (d, RE = 0·72, CVRE = 0·95) and species occurrence (e, RE = 0·94, CVRE = 1·02) for the fragmentation study (*n* = 52). Cover (proportion) data were log‐transformed and site standardized. Euclidean distance was used for splitting. Explanatory variables shown are as follows: grazing level (low, medium, high); time since fencing (years); bioregion (calcareous dunes and alluvial plains); total soil N (%); remnant patch size (small = 0·5–5 ha, medium = 5–10 ha, large >20 ha); soil pH; location in remnant (lee = leeward edge, centre = remnant centre, wind = windward edge); and organic soil C (%).

Across two independent data sets, biocrust morphogroup composition showed a stronger response to explanatory variables in MRTs than either species abundance or species occurrence, with some overlap in selected variables across the levels of data resolution (i.e. morphogroups vs. species data). Overall, degradation variables best explained and predicted morphogroup abundance, whereas specific environmental gradients explained species abundance (Fig. 1). Species occurrence responded more weakly to explanatory variables, and models had weak or no predictive power.

More specifically, the best MRT of morphogroup data from the fencing study (Fig. 1a) had relatively strong explanatory and predictive power (relative error, RE = 0·48; cross‐validated relative error, CVRE = 0·67) and separated quadrats based on grazing level, time and organic soil C (%). The best MRT classification of species abundance data from the fencing study had moderate explanatory and predictive power (Fig. 1c, RE = 0·71, CVRE = 0·80) and separated quadrats on gradients in soil pH and organic soil C (%). The best MRT classification of species occurrence data from the fencing study had weak explanatory and predictive power (Fig. 1b, RE = 0·81, CVRE = 0·96) and separated quadrats on time since fencing and total soil N (%).

The MRT of morphogroup data from the fragmentation study (Fig. 1d) had moderate explanatory power but weak predictive power (RE = 0·72, CVRE = 0·95) and separated quadrats on remnant size, location within the remnant and bioregion. The MRT of species occurrence for the fragmentation study had weak explanatory power and no predictive power (Fig. 1e, RE = 0·94, CVRE = 1·02), and quadrats were separated on remnant size only.

### Morphogroups are Useful and Informative

Overall, our study suggests that field surveys of biocrusts are more useful at the morphogroup level than at species level. Not only are morphogroups quicker and easier to survey than species, but quadrat‐scale variation in morphogroup abundance revealed compositional shifts of biocrusts in response to ecological degradation, whereas species abundance data revealed little beyond environmental influences.

### Morphogroup Indicators of Site Degradation

Overall, short mosses were indicators of more degraded sites, and tall mosses and some lichen morphogroups were indicators of less degraded sites in both studies (Table 2). In the fencing study, short moss was an indicator of intensely grazed sites (level = 3) that had been recently fenced from livestock (<3 years), while tall moss was an indicator of lightly grazed sites (levels 1 and 2) that had been fenced >3 years. When we did not distinguish between short and tall mosses in the fragmentation study*,* mosses generally were an indicator of small (degraded, high livestock density) sites. Foliose lichens were the indicator group for large and medium (less degraded, low stocking density) sites, and squamulose lichens, the indicator group for the more protected centre and leeward (eastern) side of remnants in the fragmentation study. Gelatinous and squamulose lichens were indicators of long ungrazed sites (>29 years) in the fencing study as well as thallose liverworts, black crusts and short moss.

**Table 2:** Morphogroup and species indicators identified in the best^a^ predictive multivariate regression trees (MRTs) based on abundance data, where indicator values represent group fidelity and abundance in the group, probability of group membership is shown, and the breakpoint is the value of the explanatory variable that defines the group

Study	Breakpoints	Indicator groups	Indicator value	Probability
*Fragmentation study*				
Morphogroup abundance	Large and medium sites	Foliose lichen	0·65	0·016
	Small sites	Moss	0·62	0·028
	Centre and leeward edge	Squamulose lichen	0·62	0·003
*Fencing study*				
Morphogroup abundance	Grazing level = low‐medium	Tall moss	0·85	0·001
	Grazing level = high	Short moss	0·77	0·006
	Time ≥29	Gelatinous lichen	0·78	0·001
		Squamulose lichen	0·77	0·02
		Thallose liverwort	0·73	0·02
		Short moss	0·59	0·037
		Black crust	0·47	0·003
	Time <29	Tall moss	0·70	0·04
	C ≥1·6	Tall moss	0·69	0·003
	C <1·6	Short moss	0·71	0·003
		Squamulose lichen	0·57	0·010
		Black crust	0·53	0·028
		Leafy liverwort	0·31	0·014
	Time ≥3	Tall moss	0·61	0·013
	Time <3	Short moss	0·79	0·001
*Species abundance*	pH ≥6·5	*Gemmabryum pachytheca*	0·65	0·003
		*Didymodon torquatus*	0·49	0·02
		*Fissidens megalotis*	0·44	0·02
		*Tortula atrovirens*	0·42	0·024
		*Cladia* sp.	0·38	0·017
		*Gigaspermum repens*	0·35	0·021
		*Collema coccophorum*	0·35	0·025
		*Riccia limbata*	0·25	0·035
	pH <6·5	*Triquetrella papillata*	0·78	0·001
		*Xanthoparmelia amphixantha*	0·36	0·006
	C ≥2·0	*Syntrichia antarctica*	0·83	0·004
	C <2·0	*Pseudocrossidium crinitum*	0·58	0·045
		*Triquetrella papillata*	0·63	0·011

a Best MRTs (i.e. trees with lowest CVRE) were selected through the *best subsets model selection* procedure.

## Discussion

### The Value of Biocrusts for Surveys of Dryland Integrity

Drylands are tightly controlled ecosystems (Noy‐Meir 1973) with three foci of biological activity: above‐ground plant biomass, rhizospheres and biocrusts between the plant canopies. We already have a good understanding of using plant communities as indicators of dryland degradation. While rapid assessment of rhizospheres may be impractical, sound rapid assessment techniques for biocrusts could strengthen our ability to detect degradation. Biocrusts are a primary agent in stabilizing dryland soils against erosion (Chaudhary *et al*. 2009) and are amongst the most sensitive and rapid response indicators of perturbation (Eldridge, Val & James 2011).

### The Utility of Surveying Morphogroups

Our results suggest that morphogroups are useful for assessing shifts in biocrust composition because morphogroups operate as response groups. Morphogroup abundance at the quadrat scale responded strongly to explanatory variables in MRT models for two independent data sets. These MRT models were predictive, even for the untargeted and noisier fragmentation data (which was unstratified and sampled biocrust‐free microsites covered by leaf litter). Morphogroups were useful for detecting shifts in biocrust composition in response to degradation, because morphogroups responded most strongly to degradation variables; that is, degradation variables ranked highly and were selected more often in the MRTs than other variables (Fig. 1). These results support Eldridge & Rosentreter (1999) who first suggested morphogroups as a useful tool for investigating biocrust response to ecological degradation.

In the light of our results and the strong links between biocrust composition and the ecological integrity of sites where they occur (Bowker *et al*. 2008), we argue that investing time in surveys of biocrust morphogroups is worthwhile for practitioners. Morphogroup data can provide insights into ecological integrity that would be overlooked in standard rangeland assessments (Pellant *et al*. 2000; Tongway & Hindley 2004) that use biocrust cover alone.

### Why do Morphogroups Generalize Species Response to Ecosystem Degradation?

We suggest that morphogroups summarize and generalize species’ response to degradation because species respond more similarly to degradation within morphogroups than across groups. Eldridge & Rosentreter (1999) argued that biocrust species within morphogroups respond similarly to degradation because of similarities in external morphology. Our results support this premise. We also think attributes such as structural strength and growth rate are important. A recent study from Antarctica suggests that mosses have morphological adaptations that slow desiccation rates compared to lichens, extending their metabolically active periods and their opportunities for growth (Schlensog, Green & Schroeter 2013). Lichens become brittle when dry and readily fragment (Heinken 2007), and attachment to the soil by rhizines is often weak. In contrast, many mosses grow up through trapped sediments and become embedded (Danin & Ganor 1991), providing some protection and a more secure attachment. Mosses and foliose lichens were indicators of small (degraded) and large (less degraded) sites, respectively, in the fragmentation study. If foliose lichens are more prone to physical destruction and have shorter growth periods than mosses, this may explain their comparative sensitivity to degradation in our study.

Height appears to be another determinant of morphogroup response to degradation. Tall mosses and foliose (upright) lichens were identified as indicators of sites with low livestock pressure in the fencing study (i.e. large sites and low grazing levels, respectively), while short mosses were identified as indicators of sites with high grazing levels. The tall‐statured morphogroups are more exposed to shear forces of livestock trampling, and it is unsurprising that they indicate protected sites. Similarly, squamulose lichens were indicators of sampling locations protected from sediment deposition (i.e. locations not at the windward edge of remnants). Deposition of coarse sediments at the windward edge (Duncan *et al*. 2007) would preclude slow‐growing, short‐statured species that are easily covered.

The indicator analysis differentiated morphogroups along a successional gradient of time‐since‐livestock exclusion (time). Short mosses were indicators of recently grazed sites (<3 years), while tall mosses were indicators of intermediate periods of livestock exclusion (3–29 years). Interestingly, this result mirrors a study by Hilty *et al*. (2003) who observed succession of short by tall mosses following fire. They proposed that a beneficial association between short mosses and nitrogen‐fixing cyanobacteria allowed short mosses to grow more successfully in nutrient‐depleted soils. The congruence between our study and Hilty *et al*.'s (2003) is interesting in the light of the different disturbances between the two studies.

Indicators of long ungrazed sites (>29 years) were somewhat surprising, with inclusion of gelatinous lichens (cyanolichens) and black (cyanobacterial) crusts. This conflicts with the standard view of biocrust succession where these groups are described as important colonizers (e.g. Belnap & Eldridge 2003; Langhans, Storm & Schwabe 2009) and indicators of early‐successional seres. However, other studies have observed early dominance by mosses and later dominance by cyanobacterial and algal species (e.g. Li *et al*. 2002), suggesting that the standard view of biocrust succession requires re‐evaluation. Why short mosses are also indicators of late‐successional sites (>29 years) is unclear, but suggests that further refinement of morphogroups is necessary to differentiate between early‐ and late‐successional taxa.

In addition to traits explaining the performance of morphogroups as response groups, we propose additional explanations for why species data MRTs had lower prediction and explanatory power than morphogroup MRTs. First, there are more ways many species can vary across ecological gradients than few morphogroups. This would make it more difficult to identify distinct species communities and associated ecological conditions without very large sample sizes. Second, the distribution of species may be relatively random, with different species occupying the same functional response groups. Finally, MRTs of species may be influenced by strong responses of a few species to particular conditions (despite standardization of data prior to analyses), while morphogroups are unlikely to occupy narrow ecological ranges, because they summarize the response of many species.

### What can Morphogroups Reveal about Biocrust Integrity and Ecological Integrity?

Our MRT models of morphogroup data are predictive (i.e. useful for predicting morphogroup composition from degradation variables), which means that there is potential to infer the degree of ecological degradation from the prevalence of different morphogroups compared to ecological reference sites, or to track changes in ecological integrity over time by monitoring shifts in morphogroup prevalence. For example, in our study area, an increase in short mosses and a decrease in foliose and squamulose lichens over time may inform rangeland managers that livestock pressure should be reduced, particularly if these groups in turn had identifiable effects on ecosystem processes, such as resistance to erosion. While some species will fail to fit neatly within groups and changes in species composition may go undetected when using morphogroups (Eldridge & Rosentreter 1999), our study supports the general utility of this scheme. Morphogroups appear to be an efficient coding of species response to ecological degradation. This is similar to plant life‐forms that effectively characterize plant community response to degradation (McIntyre, Lavorel & Tremont 1995) and feeding guilds of river invertebrates that capture biotic response to changed water quality (Osborne, Davies & Linton 1980).

### Reflections on Multivariate Regression Trees

Our development of a best subsets model selection procedure (code in Appendix S3, Supporting information) improves the utility of the MRT method. The best subsets method provided insight into important predictor variables and improved model robustness and predictive capacity.

### Conclusion

Biocrust morphogroups are an informative tool for vegetation managers who require rapid‐survey data that will provide insight into the ecological integrity of drylands. MRTs combined with our novel best subsets method gave confidence that minimal information was lost when using morphogroups, a reduced set of biotic classes that enhance survey speed. Morphogroups responded strongly to degradation variables, suggesting that they provide an efficient coding of biocrust species response to ecological degradation and are useful indicators of dryland degradation.

## Supporting information


**Appendix S1.** Species frequency and morphogroup classification.

**Appendix S2.** Multivariate regression tree (MRT) analyses.

**Appendix S3.** Cross‐validation procedure.

**Appendix S4.** Best subset model selection procedure.

**Appendix S5.** Null models.
