# Supplementary material for: Biocrust morphogroups provide an effective and rapid assessment tool for drylands
Source: J Appl Ecol. 2014 Oct 1;51(6):1740–9. doi: 10.1111/1365-2664.12336 (PMC4286204; doi:10.1111/1365-2664.12336)
Supplement: Supplementary file 1 — Appendix S1. Species frequency and morphogroup classification. [file JPE-51-1740-s001.docx]

**Appendix S1.** Species frequency and morphogroup classification

Species frequency and morphogroup classification^a,b^ (species occurring > 5 % quadrats in fragmentation study and all species in the taxonomically resolved fencing study)

| **Morphogroup** | **Species** | **Fencing study**  **(n= 61)** | **Fragmentation study**  **(n = 52)** |
| --- | --- | --- | --- |
| Black crust (cyanobacteria and algae, visible as dark soil colouration)^c^ | | | |
|  | | 27.0 | NA |
| Gel lichens (cyano-lichens, gel-like when wet)^c^ | | | |
|  | *Collema coccophorum* | 27.0 | 42.6 |
|  | *Lempholemma chalazanum.* | 17.5 |  |
|  | *Synalissa symphorea* |  | 6.6 |
| Crustose lichens (crust-like growth, tightly attached to soil) | | | |
|  | *Aspicilia contorta* | 4.8 | 32.8 |
|  | *Diploschistes thunbergianus* | 3.2 | 16.4 |
|  | *Lecinora* sp aff. | 3.2 |  |
|  | *Paraporpidia glauca* | 3.2 |  |
|  | *Psora crystallifera* | 6.3 | 18.0 |
| Squamulose lichens (scale-like thallus) | | | |
|  | *Catapyrenium* sp*.* |  | 8.2 |
|  | *Cladonia* sp*.* | 30.2 | 27.9 |
|  | *Endocarpon pusillum* | 11.1 |  |
|  | *Endocarpon simplicatum* | 14.3 |  |
|  | *Endocarpon* sp*.* |  | 26.2 |
|  | *Placidium squamulosum* | 12.7 | 24.6 |
|  | *Psora decipiens* | 3.2 | 21.3 |
|  | *Trapelia crystallifera* | 3.2 | + |
| Foliose lichens (leaf-like, flattened thallus) | | | |
|  | *Cladia beaugleholei* | 6.3 | 9.8 |
|  | *Cladia muelleri* | 6.3 |  |
|  | *Siphula coriacea* | 4.8 | 6.6 |
|  | *Xanthoparmelia amphixantha* | 22.2 | 21.3 |
|  | *Xanthoparmelia reptans* | 27.0 | 11.5 |
|  | *Xanthoparmelia pulla* | 6.3 | + |
|  | *Xanthoparmelia willisii* | 7.9 |  |
| Fruticose lichens (shrub-like, branched thallus) | |  |  |
|  | *Cladia aggregata* | 7.9 | + |
| Short mosses (species mean height <15 mm)** | | | |
|  | *Acaulon integrifolium* | 4.8 |  |
|  | *Aloina sullivaniana* |  | 11.5 |
|  | *Barbula unguiculata* | 6.3 |  |
|  | *Bartramia nothostricta* | 1.6 |  |
|  | *Bryobartramia novae-valesiae* | 1.6 |  |
|  | *Bryum argenteum* | 11.1 | 19.7 |
|  | *Ceratodon purpureus* |  | + |
|  | *Crossidium geheebii* |  | + |
|  | *Didymodon torquatus* | 50.8 | 55.7 |
|  | *Eccremidium pulchellum* | 7.9 | + |
|  | *Entosthodon subnudus* |  | 23.0 |
|  | *Fissidens megalotis* | 38.1 | 32.8 |
|  | *Fissidens taylorii* | 9.5 | + |
|  | *Gemmabryum pachytheca group* | 57.1 | 62.3 |
|  | *Gigaspermum repens* | 28.6 | 37.7 |
|  | *Goniomitrium acuminatum* ssp. *enerve* | 1.6 | + |
|  | *Microbryum* sp. aff | 1.6 |  |
|  | *Microbryum davallianum* |  | + |
|  | *Microbryum starkeanum* |  | 8.2 |
|  | *Phascopsis rubicunda* | 1.6 |  |
|  | *Phascum robustum* var*. crassinervium* | 6.3 |  |
|  | *Pottia scabrifolia* | 1.6 |  |
|  | *Pottia* sp. |  | 11.5 |
|  | *Pseudocrossidium hornschuchianum* | 9.5 | 14.8 |
|  | *Pterygoneurum ovatum* | 3.2 | + |
|  | *Stonea oleaginosa* |  | 11.5 |
|  | *Tetrapterum cylindricum* | 4.8 |  |
|  | *Tortula atrovirens* group | 41.3 | 49.2 |
| Tall mosses (species mean height >15 mm) | | | |
|  | *Barbula calycina* | 46.0 | 29.5 |
|  | *Campylopus introflexus* | 3.2 | + |
|  | *Pseudocrossidium crinitum* | 41.3 | 14.8 |
|  | *Rosulabryum campylothecium* | 17.5 | 13.1 |
|  | *Rosulabryum capillare* | 20.6 | 11.5 |
|  | *Syntrichia antarctica* | 42.9 | 9.8 |
|  | *Triquetrella papillata* | 69.8 | 23.0 |
| Thallose liverworts (strap-like, flattened thallus) | | | |
|  | *Riccia limbata* | 17.5 | 16.4 |
|  | *Riccia* sp. | 7.9 | 16.4 |
|  | *Riccia spongiosula* | 1.6 |  |
| Leafy liverworts (leafy plants) | | | |
|  | *Cephaloziella exiflora* | 1.6 | 6.6 |
|  | *Fossombronia* sp*.* | 6.3 | 16.4 |

^a^Taxa confidently identified to species level in the fragmentation study and recorded with a frequency < 5 % across quadrats are noted (+)

^b^Nomenclature generally follows Klazenga (2013) for mosses, McCarthy (2006) for liverworts and McCarthy (2013) and J. Elix (pers. comm.) for lichens.

^c^Black crust (cyanobacterial crust) and gel lichen morphogroups were not recorded and mosses were recorded as one morphogroup (moss) in the fragmentation study
