# Supplementary material for: Biocrust morphogroups provide an effective and rapid assessment tool for drylands
Source: J Appl Ecol. 2014 Oct 1;51(6):1740–9. doi: 10.1111/1365-2664.12336 (PMC4286204; doi:10.1111/1365-2664.12336)
Supplement: Supplementary file 2 — Appendix S2. Multivariate regression tree (MRT) analyses. [file JPE-51-1740-s002.docx]

# Appendix S2. Multivariate regression tree (MRT) analyses

MRTs are a constrained analysis that repeatedly splits the assembled set of quadrat data into two nodes or groups that represent a distinct community composition, with each group defined by a threshold value of an associated environmental variable (De’ath 2002). The relative error (RE) of a tree is the error of the final tree relative to the error of the initial, unsplit tree and is the inverse of its explanatory power or R^2^. In common with most evaluation measures based on training data (i.e. the data used to fit the model), the RE gives an overly “optimistic” impression of the predictive performance of a tree. We were most interested in predictive performance, so used the cross-validated relative error (CVRE) to select the best MRT. The CVRE represents the capacity of the tree to predict community composition for new quadrats. Calculation of the CVRE is based on a repeated random sub-sampling cross-validation, where number of cross-validations ("xval") can be specified and controls the proportional allocation of sites to training and test (evaluation) sets (eg in mvpart with xval = 10, 90% of the data are used for training and the remaining 10% for testing, and this is repeated 10 times, where each time data are randomly allocated to train and test groups). The CVRE is the average test error over the chosen number of cross-validations. We repeated the cross-validation 100 times (in mvpart, xvmult=100) to stabilise variability in CVRE estimates due to the random cross-validation; the mvpart package then estimates the mean CVRE. A CVRE of 0 indicates perfect prediction and a CVRE >=1 indicates no predictive power. The depth (number of splits) of the trees was selected by finding that depth that fitted the best predictive tree in the cross-validation (xv="min" in mvpart).

Because MRTs are a relatively novel method we compared results with non-metric multidimensional scaling (NMDS) for BSC community data using Bray Curtis distance and vector fitting. As the NMDS provided similar results we do not discuss these results further.
