# Supplementary material for: Biocrust morphogroups provide an effective and rapid assessment tool for drylands
Source: J Appl Ecol. 2014 Oct 1;51(6):1740–9. doi: 10.1111/1365-2664.12336 (PMC4286204; doi:10.1111/1365-2664.12336)
Supplement: Supplementary file 3 — Appendix S3. Cross‐validation procedure. [file JPE-51-1740-s003.docx]

**Appendix S3.** Cross-validation procedure

We kept all data for any one site in the same cross-validation fold (i.e. the three quadrats per site for the fencing study and two-to-three quadrats per site for the fragmentation study). This means that estimates of predictive performance are not inflated by having quadrats that belong to the same site spread across training and test sets. Otherwise, predictive performance might be inflated if the quadrats within sites were more similar than quadrats across sites. More specifically, we arranged the quadrats into 10 groups, with data from 2 to 3 sites in each group. We could have chosen a cross-validation with more groups (number of groups = number of sites) but preferred the harder test of fewer groups (harder because the model is fitted to a smaller dataset). Microsite and Location were retained in the models as explanatory variables for the fencing and fragmentation study respectively.
