# Supplementary material for: Biocrust morphogroups provide an effective and rapid assessment tool for drylands
Source: J Appl Ecol. 2014 Oct 1;51(6):1740–9. doi: 10.1111/1365-2664.12336 (PMC4286204; doi:10.1111/1365-2664.12336)
Supplement: Supplementary file 4 — Appendix S4. Best subset model selection procedure. [file JPE-51-1740-s004.docx]

**Appendix S4.** Best subsets model selection

During model development we discovered that MRTs derived from a large set of explanatory variables sometimes had a higher CVRE (lower prediction) than MRTs derived from a subset of variables. This result indicated the model fitting could be “locked” into building a tree with a less than optimal predictive performance for the dataset; we think this may happen when explanatory variables higher in the tree resulted in “worse” splits (i.e. groups with higher impurity) lower in the tree, than for trees built without these distracting variables. To address this issue we developed code to fit many models on subsets of predictors, to identify the most predictive model. Following the regression literature, we call this *best subsets* *model selection* and iteratively build many MRTs, fitted to every possible subset and combination of explanatory variables (from 3 variables to the full number). We ran this best subsets procedure for each dataset and extracted the MRTs with the lowest mean CVRE and their associated explanatory variables for each dataset. Results were consistent between the top ten trees for each dataset, so we present the *best MRT* (i.e. lowest CVRE) for each dataset only.

### **Code for best subsets model selection procedure**

run.many.models <- function(predictor.vec, response.cols, data.df){

#predictor.vec - names of predictors

#response.cols - as text string eg "fenceMG3[,c(34:43)])"

#data.df - the dataframe you're using as text string- eg "fenceMG3"

all.combs <- list() #make an empty list

n <- length(predictor.vec)

for(i in 3:n){

new.lot <- combn(n, i, simplify=F) #make list with as many elements as there are combinations of i from n

all.combs <- c(all.combs, new.lot) #update the list.

}

call.ls <- list()#make a list that pulls out the names of predictors and presents them as a text string for making a function call to mvpart

for (i in 1:length(all.combs)){

preds <- predictor.vec[all.combs[[i]]]

len <- length(preds)

if(len > 1){

x.call <- preds[1]

for (j in 2:len){

x.call <- paste(x.call, "+", preds[j])

}

}

else { x.call <- preds }

call.ls[[i]] <- x.call

}

splitvars <- list() # make empty list and matrix for results

errors <- matrix(nc=2, nr=length(all.combs))

colnames(errors) <- c("error", "cverror")

markers <- seq(1, length(all.combs), by=50)

results<- list() #sort results – to view best models

sorted.errors <- errors[order(errors[,3]),]

results$sorted.errors <- sorted.errors

results$sorted.calls <- call.ls[sorted.errors[,1]]

results$sorted.splits <- splitvars[sorted.errors[,1]]

return(results) #sends the results to the model object you name when you call this function

}
