# Supplementary material for: Biocrust morphogroups provide an effective and rapid assessment tool for drylands
Source: J Appl Ecol. 2014 Oct 1;51(6):1740–9. doi: 10.1111/1365-2664.12336 (PMC4286204; doi:10.1111/1365-2664.12336)
Supplement: Supplementary file 5 — Appendix S5. Null models. [file JPE-51-1740-s005.docx]

**Appendix S5.** Null models

To investigate whether results of morphogroup analyses were somehow associated with modelling a reduced number of entities (morphogroups compared with species) we constructed null models where species were randomly allocated to ten groups (ten, because there were ten morphogroups). MRT model selection was then repeated in the same way as for morphogroup analyses. We refer to the groups formed in these analyses as randomgroups. Null models for randomgroups (fencing study) had both explanatory and predictive power. The mean relative error and cross-validated relative error for the most predictive trees based on 100 randomisations of the data was moderate (RE=0.67, StDev=0.10; CVRE=0.82, StDev=0.03). Quadrats were separated based on soil pH. Much of the variation in randomgroup composition explained by the model and all variation predicted by the model was due to variation between groups arising from assignment of the dominant species *Triquetrella papillata* to a randomgroup. When null models were run without this species they had no predictive power (CVRE=1.00, StDev=0.05) and slightly higher relative error (0.71, StDev=0.15) and quadrats were separated on soil organic C (%) and total N (%). Because explanatory variables in these null models are the same as for MRTs of species abundance, we believe the null model explains some variation in randomgroup composition due to assignment of common species. Another way to interpret this interesting test is that it shows that the Morphogroups are not only explaining a ‘significant’ component of variation, but also that they are qualitatively different in the set of variables that they respond to.
